# Clinical Applications and Outcome of Proximally Based Medial Gastrocnemius Muscle Flap

**DOI:** 10.29252/wjps.9.1.22

**Published:** 2020-01

**Authors:** Muhammad Saaiq, Farid Ullah Khan Zimri

**Affiliations:** Department of Plastic Surgery and Orthopedics, National Institute of Rehabilitation Medicine (NIRM), Islamabad, Pakistan

**Keywords:** Gastrocnemius muscle, Flap, Pretibial, Tibia, Total knee arthroplasty

## Abstract

**BACKGROUND:**

Gastrocnemius muscle flap has been in vogue for approximately five decades. The current study was carried out to document the indications and outcome of proximally based medial gastrocnemius muscle flap in our patients.

**METHODS:**

This case series was conducted in Department of Plastic Surgery and Orthopedics, National Institute of Rehabilitation Medicine (NIRM), Islamabad, Pakistan during 3 years. It included all patients who were managed with proximally based medial gastrocnemius muscle flap for various indications.

**RESULTS:**

There were 31 patients with 24 (77.41%) males and 7 (22.58%) females. The age ranged between 16- and 53 years (mean: 27.47±10.33 years). The indications for gastrocnemius muscle flap included traumatic defects with exposed tibia/ knee joint (n=20; 64.51%), prophylactic coverage of megaprosthesis employed for knee joint reconstruction (n=9; 29%), excisional defect of cutaneous squamous cell carcinoma with exposed tibia (n=1; 3.22%), and salvage of infected total knee arthroplasty (n=1; 3.22%). The hospital stay was 7-16 days (mean: 12.41±2.87 days). The flap survival in our series was 100%. There was partial skin graft in two patients (n=2; 6.45%).

**CONCLUSION:**

Gastrocnemius muscle flap was a quick, easy and reliable coverage tool for small to moderate sized defects around the knee, the proximal third of the tibia as well as coverage of prosthesesis employed for knee arthroplasty. Inclusion of 2-4 cm tendon enhances the flap dimension without causing any additional morbidity.

## INTRODUCTION

Pretibial defects of the proximal third of the leg and soft tissue defects in and around the knee may result from a variety of causes. For instance, trauma, burns, tumor resection and release of post-burn contractures of the knee.^1^ All these defects are manageable with medial gastrocnemius flap. Moreover, several uses of gastrocnemius flap have emerged over time in association with total knee arthroplasty, while in this latter scenario, it may be employed for prophylactic muscle coverage of the large implants or a rescue muscle coverage of the exposed or infected implants.^2^^-^^4^

Gastrocnemius muscle flap has been in vogue for approximately five decades. It still continues to enjoy its popularity as a viable coverage option even in the present day of highly advanced plastic surgery. Each head of the muscle has a reliable and consistent axial blood supply through a single dominant vascular pedicle arising from sural artery in the popliteal fossa. Flap elevation is technically straightforward with relatively easy dissection.^5^^-^^8^

The flap is sturdy and lacks any significant donor functional impairments. The medial gastrocnemius is larger than the lateral one, with longer vascular pedicle and greater arc of rotation. Hence, it is more frequently employed. The lateral gastrocnemius carries the additional risk of damaging the peroneal nerve, while it turns around the neck of the fibula.^5^^-^^8^ The current study was carried out to document the indications and outcome of proximally based medial gastrocnemius muscle flap in our patients.

## MATERIALS AND METHODS

This descriptive study of case series was carried out at the Department of Plastic Surgery and Orthopedics, National Institute of Rehabilitation Medicine (NIRM), Islamabad, Pakistan over a period of three years from July 1, 2015 to June 30, 2018. Informed consent was taken from the patients. The study was carried out in accordance with the Declaration of Helsinki of 1975, as revised in 2008 and anonymity of the participants was guaranteed. Informed consent was taken from all the patients.

We employed convenience sampling technique. We included all adult patients of either gender who presented with defects of small to medium size around the knee, proximal third of the tibia or cases where coverage of megaprostheses was needed. Our exclusion criteria included children <14 years, simple defects surmountable to skin grafts only, large size defects not manageable with gastrocnemius flap and injured muscle pedicle. Initial clinical evaluation was made by a thorough history, physical examination and ancillary investigations. 

The patients were hospitalized for definitive wound management and reconstruction under spinal or general anesthesia. The defects of traumatic origin were initially managed in a standard fashion. All devitalized tissues were thoroughly excised and any associated fractures fixed. Initial temporization of the contaminated wounds was done with Vacuum assisted closure (VAC) in selected cases. Cases where the wound bed was clean, immediate flap coverage was done. 

We undertook flap elevation under tourniquet control, with patient in supine position. The knee was slightly flexed and the lower limb externally rotated. The defect size assessed for the adequacy of muscle coverage. For medial gastrocnemius, incision was made 2 cm behind the medial border of tibia extending from the poplitial fossa to below mid-calf level. Incision was deepened to deep fascia and medial head of gastrocnemius was identified and separated from underlying soleus muscle. 

Distal end of the muscle was sharply divided from the Achilles tendon taking care to include 2-4 cm portion of tendon. It was then divided and separated from lateral gastrocnemius at the midline raphe. Care was taken to preserve the underlying vital structures such as sural nerve, short saphenous vein and plantaris tendon. Muscle was then transposed on to the defect. Split thickness skin grafting of muscle was done where indicated. The limb was immobilized for one week postoperatively. 

During immobilization, limb elevation over a pillow was ensured to prevent edema. The flap was checked on the fifth postoperative day to establish flap survival or loss, any necrosis of skin graft and infection. The data were analyzed statistically using statistical package for social sciences (SPSS, Version 17, Chicago, IL, USA) and various descriptive statistics were employed to calculate the objectives. The primary outcome measure was flap survival or otherwise, while the secondary outcome measures included skin graft take, duration of hospital stay and any complications.

## RESULTS

There were 31 patients with 24 (77.41%) males and 7 (22.58%) females. The age ranged from 16 to 53 years with a mean of 27.47±10.33 years. The muscle flap was resurfaced with split thickness skin grafts among 21 patients (67.74%), whereas among the remainder 10 patients (32.25%), the flap was employed under intact skin cover. Seven defects (22.58%) were small sized (i.e. ≤25cm^2^), whereas 24 (77.41%) were medium sized (i.e. ≥25 cm^2^ and ≤105 cm^2^). The indications for gastrocnemius muscle flap included traumatic defects with exposed tibia/knee joint (n=20; 64.51%), prophylactic coverage of megaprosthesis employed for knee joint reconstruction (n=9; 29%), excisional defect of cutaneous squamous cell carcinoma with exposed tibia (n=1; 3.22%), and salvage of infected total knee arthroplasty (n=1; 3.22%). 

**Fig. 1:**
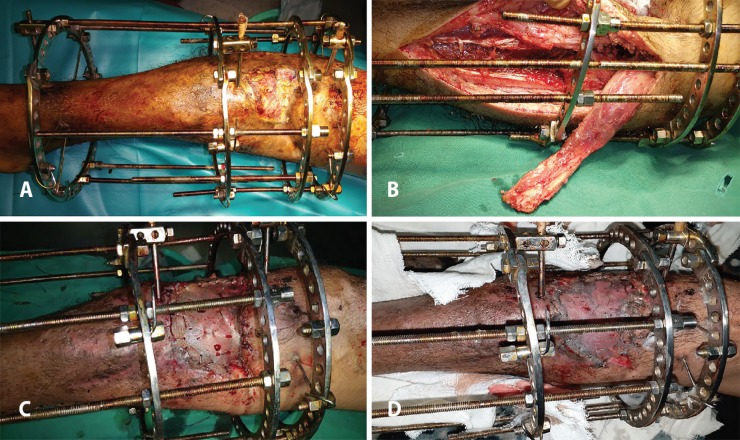
**(A): **A 17 years old boy with post-traumatic pretibial defect. The associated fracture has been stabilized with an Ilizarov fixator. **(B)**: Intraoperative photograph of the same patient as in Figure 1A following elevation of medial gastrocnemius muscle flap with the fixator in situ. **(C): **Medial gastrocnemius muscle flap with split thickness skin graft employed for reconstructing the defect shown in Figure A and B. **(D**): Same patient as in Figure A, B and C one week after reconstruction, showing flap survival and good graft take

The hospital stay was 7-16 days with a mean stay of 12.41±2.87 days. The flap survival in our series was 100%. We encountered partial skin graft in two patients (n=2; 6.45%). Figures 1 (a) through 2 (e) show two illustrative cases included in the study. Figure 3 denotes to medial gastrocnemius muscle flaps elevated through a separate incision and Figure 4 reveals knee joint reconstruction and elevated medial gastrocnemius muscle flaps too.

**Fig. 2:**
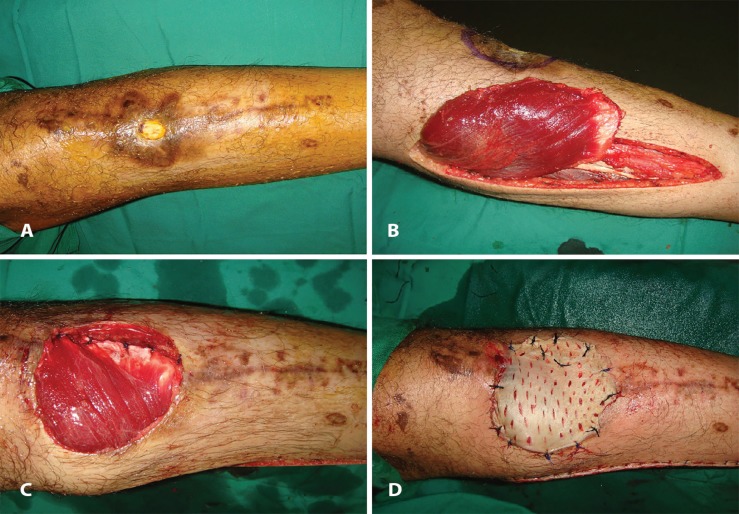
**(A):** A left sided pretibial defect post automobile accident in a 27 years old male. **(B):** Same patient as in Figure 2A with elevated medial gastrocnemius muscle flap. **(C):** Intraoperative photograph of the same patient as in Figure 2A and B, following inset of the muscle flap in the defect. **(D):** Intraoperative photograph of the same patient as in Figure 2A, B and C, Medial gastrocnemius muscle flap with split thickness skin graft employed for reconstructing the defect

**Fig. 3:**
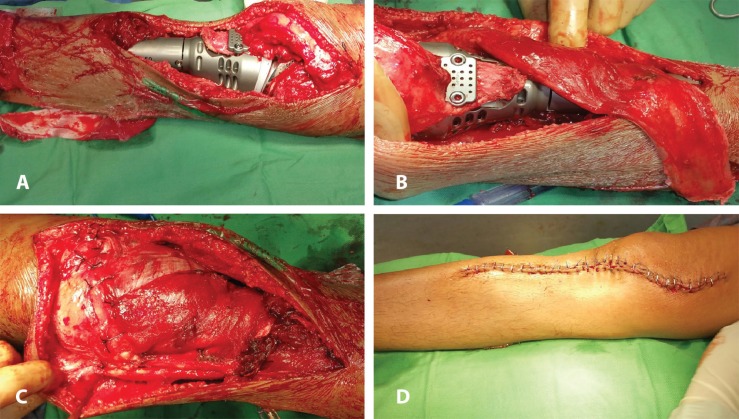
**(A):** An 18 years old female with osteosarcoma of proximal tibia following curative tumour excision and knee joint reconstruction with megaprosthesis. Medial gastrocnemius muscle flap has been elevated through a separate incision. **(B):** Patient as in Figure 3A. The medial gastrocnemius muscle flap has been elevated 4 cm tendon to ensure adequate coverage of the magaprosthesis. **(C):** Intraoperative photograph of the same patient as in Figure 3A, and B, following inset of the muscle flap to cover the prothesis. **(D):** Completion photograph of the same patient as in Figure 3A, B and C

**Fig. 4:**
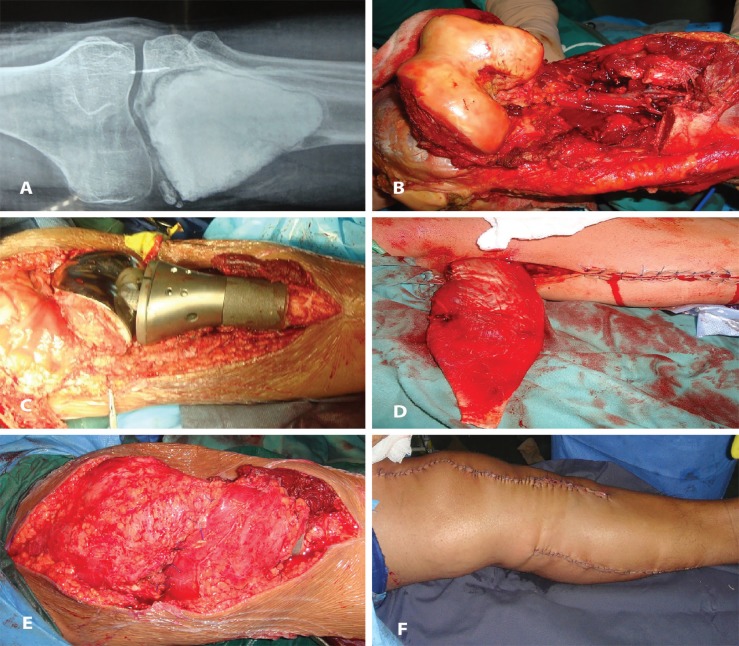
**(A):** A 28 years old male with giant cell tumour of the left tibia. **(B): **Same patient as in Figure 4A the excisional defect following curative resection of the tumor. **(C): **Same patient as in Figure 4A and B knee joint reconstruction with megaprosthesis. **(D): **The medial gastrocnemius muscle flap has been elevated with 4 cm tendon to provide for adequate coverage of the magaprosthesis. **(E):** Following inset of the muscle flap to cover the prothesis. **(F): **Completion photograph of the same patient as in Fig 4A through F

## DISCUSSION

Gastrocnemius represents one of the most robust and reliable muscle flaps. One peculiar characteristic of this muscle is that each of the medial and lateral bellies serves as a complete muscle flap on its own, each having its independent blood supply by the medial and lateral sural arteries. These arteries arise from the posterior aspect of the popliteal artery approximately 1cm proximal to the knee joint line. The length of each vascular pedicle is approximately 5 cm and courses longitudinally along the muscle belly.^8^^-^^11^

The medial head of the gastrocnemius muscle in adult measures 15-20 cm in length and 8 cm in width. The lateral head of the gastrocnemius muscle measures 12-17 cm in length and 6 cm in width. The medial belly is more commonly used as it is larger and better developed than the lateral one. Also, the excursion of the lateral belly is restricted by the fibula.^8^^-^^11^ Majority of our patients had open tibial fractures with soft tissue loss. These defects pose formidable issues for reconstructive surgeons. 

The reconstructive requirements in such defects included coverage of the exposed vital structures such as the bones or joint, obliteration of the dead space and possible eradication any associated infection. Early coverage is often needed. The gastrocnemius muscle flap beautifully meats all these reconstructive requirements in most instances. The muscle flap brings robust blood supply to the affected area which in turn expedites tissue healing and infection control. It also perfuses the fracture site with myokines and progenitor cells that differentiate into osteocytes. This results in better bone healing and averts the chance of fracture nonunion or delayed union.^11^^-^^13^

In nine of our patients, we employed the medial gastrocnemius muscle flap for prophylactic coverage of megaprostheses employed for knee reconstruction following excision of various bone tumours. The goal was to ensure complete coverage of the prosthesis and direct suturing of its proximal edge to the edges of the knee capsule and patellar tendon. To our knowledge, we are the first to report this procedure from Pakistan. The modular endoprostheses are currently used by majority of the surgeons across the globe. 

Their beauty is that their standard sized component sets with varying lengths are readily available for use in reconstructing any given defects of the bones around knee. The various components are readily assembled in different combinations to reconstruct the large skeletal defect and achieve a functioning knee joint.^9^^,^^10^^,^^14^^,^^15^ In one patient, we employed the flap to successfully salvage an infected total knee arthroplasty. The management of infected total knee arthroplasty poses a considerable challenge. 

The infection rate in patients having a primary total knee arthroplasty was approximately 2% and may be higher with host factors such as an underlying diabetes mellitus. The associated exposure of bone or implant may sometimes lead to limb amputation. Hence, it warrants robust management. Although several techniques have been developed for providing coverage of the exposed prosthesis, the medial gastrocnemius flap continues to be the preferred method of soft tissue reconstruction. The flap is reported to salvage an infected prosthesis in up to 92% of the patients.^16^^-^^19^

We had no flap failure in the current study. Daigeler *et al.*^9^ reported on complete flap loss in one case in a large case series entailing 218 patients. The published literature has highlighted several possible causes of flap failure. For instance, direct injury of the sural vessels and kinking or compression of the vascular pedicle during transposition. There is also risk of injury during resection of the distal femur particularly at the time of popliteal exploration. The sural vessels may be mistaken for geniculate branches. It is prudent to leave generous amount of fat and fascia along the pedicle to avoid such undesirable injury to pedicle.^4^^,^^9^^,^^20^^,^^21^

We usually harvested the flap with 2-4 cm tendinous part of the Achilles tendon. This favorably increased the dimension in the most needed leading part of the tendon. This helped to adequately cover even moderate size defects. This strategy particularly helped to completely cover the megaprostheses in knee reconstruction cases. We recommend this modification as a useful one. Gastrocnemius muscle flap was a quick, easy and reliable coverage tool for small to moderate sized defects around the knee, the proximal third of the tibia as well as coverage of prosthesesis employed for knee arthroplasty. Inclusion of 2-4 cm tendon enhanced the flap dimension without causing any additional morbidity.

## CONFLICT OF INTEREST

The authors declare no conflict of interest.
